# Nanopatterning on silicon surface using atomic force microscopy with diamond-like carbon (DLC)-coated Si probe

**DOI:** 10.1186/1556-276X-6-518

**Published:** 2011-09-02

**Authors:** Xiaohong Jiang, Guoyun Wu, Jingfang Zhou, Shujie Wang, Ampere A Tseng, Zuliang Du

**Affiliations:** 1 Key Laboratory of Special Functional Materials of Ministry of Education, Henan University, Kaifeng 475004, People's Republic of China; 2 Ian Wark Research Institute, University of South Australia, Mawson Lakes SA 5095, Australia; 3 School of Engineering of Matter, Transport and Energy, Arizona State University, Tempe, AZ 85287-6106, USA

## Abstract

Atomic force microscope (AFM) equipped with diamond-like carbon (DLC)-coated Si probe has been used for scratch nanolithography on Si surfaces. The effect of scratch direction, applied tip force, scratch speed, and number of scratches on the size of the scratched geometry has been investigated. The size of the groove differs with scratch direction, which increases with the applied tip force and number of scratches but decreases slightly with scratch speed. Complex nanostructures of arrays of parallel lines and square arrays are further fabricated uniformly and precisely on Si substrates at relatively high scratch speed. DLC-coated Si probe has the potential to be an alternative in AFM-based scratch nanofabrication on hard surfaces.

## Introduction

Nanolithography is crucial to realize a size below 100 nm for nanoelectronic devices and high density recording systems [1,2]. Apart from conventional, expensive optical and electron beam lithography [1,2], scanning probe microscopy (SPM), especially scanning tunneling microcopy (STM) and atomic force microscopy (AFM)-based nanofabrication technique have been intensively studied. To date, several SPM-based nanolithography techniques have been developed including local oxidation of the surfaces of silicon and metals [3-5], dip-pen method [6,7], thermal-mechanical writing [2,8], and mechanical/electrochemical modification of a material's surface [9-11]. In recent years, although the uncertainty (drift, hysteresis, creep for AFM) will limit its application in nanostructure fabrication at large scale, AFM-based scratch nanolithography has emerged as a promising technique for nanofabrication because of its simplicity, versatility, reliability, and operation in ambient conditions [3-12]. It is also expected to fabricate nanostructure at a large scale with combination of nano-imprint system. AFM scratching technique takes advantage of the ability of moving a probe over a sample surface in a controllable way. By controlling the applied normal force (*F*_n_) between a probe and a sample surface, trenches or grooves with depths from a few to tens of a nanometer and widths from tens to hundreds of nanometers can be fabricated on both soft and hard substrates, involving polymer [13], silicon [14], oxides [15], magnetic metals, and semiconductor materials [16]. This technique thus has the potential to benefit the fabrication of nanoelectronic devices such as nanodots [17], nanowires [18], and single electron devices [1]. For example, the patterning on sapphire substrate by AFM-based nanolithography can reduce the dislocation density for III-nitride based light emitting diodes [19-21].

Previous reports in AFM-based scratch nanolithography has focused on making different nanodevices and nanosystems, in which a Si or Si_3_N_4 _tip with a typical radius of less than 20 nm was used, and the scratch was processed mainly on flexible polymer substrates [1,11,18]. To scratch on a hard Si surface, an AFM tip with high wear resistance has to be used. Recently, SPM scratch nanopatterning on a Si surface was investigated by several groups [22-24], the tips, however, were coated exclusively with diamond, which is costly. According to our knowledge, AFM scratching using diamond-like carbon (DLC)-coated probe has not been reported. DLC film is an amorphous film, and its surface is very smooth. Because of its high hardness and high elastic modulus, low coefficient of friction, wear and good tribological property, it is suitable as a wear-resistant coating [25]. From the preparation point of view, the cost for DLC films is much cheaper than that of diamond, and the commercial DLC-coated tip can easily be obtained. In the present study, we explored the potential of this economic probe in fabricating nanopatterns on hard silicon surface. The scratch characteristics were investigated and correlated to the scratch parameters. More complex nanostructures such as line and square arrays were further fabricated using a DLC-coated tip on a silicon substrate.

## Experiment

**Figure 1:**
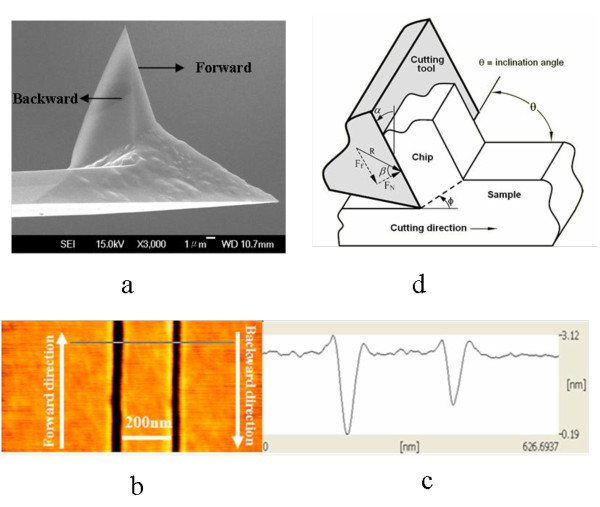
**SEM and AFM images**. **(a) **SEM image of DLC-coated triangular pyramid tip, together with the schematic of the scratch direction. **(b) **The AFM images of the typical grooves scratched at 10 μN of tip force, 1 μm/s of speed. **(c) **The cross-section profiles of the grooves at the position as indicated by the line. **(d) **Schematic of oblique cutting, inclination angle *θ *defined as the angle between the directions of scratching and cutting face.

The silicon surface selected was polished single crystal p-type Si(100). Before scratching, the sample was cleaned thoroughly by sonication in acetone and ethanol, respectively and then rinsed with deionized water. The centerline average roughness (*R*_a_) and the maximum roughness (*R*_max_) of the sample surfaces calculated from 2.0 × 2.0 μm^2 ^topographic AFM images were less than 0.14 and 1.19 nm, respectively. Scratch experiments and AFM imaging were carried out in an ambient condition using a vector scan method. Firstly, the sample surface approached the commercial DLC tip in the *Z*-direction until the tip contacting with the sample surface under a preset load. Then, the feedback loop was closed and the PZT actuator drove the sample moving in the *x*- and *y*-direction, and nano grooves were scratched under the constant set normal force, scan speed, and repeated times for scratch [26]. Finally, the AFM tip was lifted to the original height, and the grooves' structure was characterized by AFM after the nanofabrication. The DLC-coated Si probe for scratching has been sharpened into a triangular pyramid. A scanning electron microscope (SEM) image was shown in Figure 1a. The thickness of the DLC layer is approximately 15 nm, and the tip radius of curvature is about 15 nm. The spring constant of the tip is 48 N/m, indicating the tip has a high wear resistance, which aims to keep the sharpness of the tip unchanged during scratching. The AFM images of the surfaces before and after scratch were measured in contact mode using a Si_3_N_4 _tip, which has a low spring constant of about 0.02 N/m to avoid additional damage to the sample surface.

## Results and discussion

Two scratch directions, forward and backward, were selected to scratch the Si surface. As illustrated in Figure 1a, forward scratch has the sharp cutting edge along the scratch direction, while the backward scratch uses the flat cutting edge facing the scratch direction. The influence of scratch direction on the size of the scratched geometry was initially investigated. The AFM images of the typical grooves in both directions are given in Figure 1b, along with the corresponding cross-section profiles of the grooves as shown in Figure 1c, where the scratch was performed at 1 μm/s of the scratching speed and 10 μN of the applied normal force. The cross-section profiles are V-shaped in both scratch conditions. However, the depth of the groove generated in the forward scratch is clearly deeper than that in the backward direction, as seen in Figure 1c. This could be attributed to the sharpness of the tip in the forward direction, where the effective normal force was higher and a deeper groove was thus produced [27]. A similar phenomenon was observed in the investigation of AFM scratch on Si surface with diamond-coated Si tip [22-24,28]. The pyramidal tip has three scratching faces as shown in Figure 1d, the inclination angle, *θ*, is defined as the angle between the directions of scratching and cutting face. In the case of the backward direction, the tip scratch face is perpendicular to the scratch direction, i.e., the inclination angle, *θ*, equals 90°, which satisfies the requirement to become orthogonal cutting. The protuberances are created evenly along two sides of the grooves. On the other hand, if scratching is in the forward direction, the scratching face is composed of two inclination angles, i.e., one is -30° and the other is 30°. As a result, the protuberances are squeezed evenly onto the two sides of the groove scratched. Since the 30° or -30° inclination angle provides much more favorable stress states to squeeze the materials onto the two sides as compared with that of the 90° inclination angle, the protuberances created in forward scratching is more or larger than that of the backward direction.

**Figure 2:**
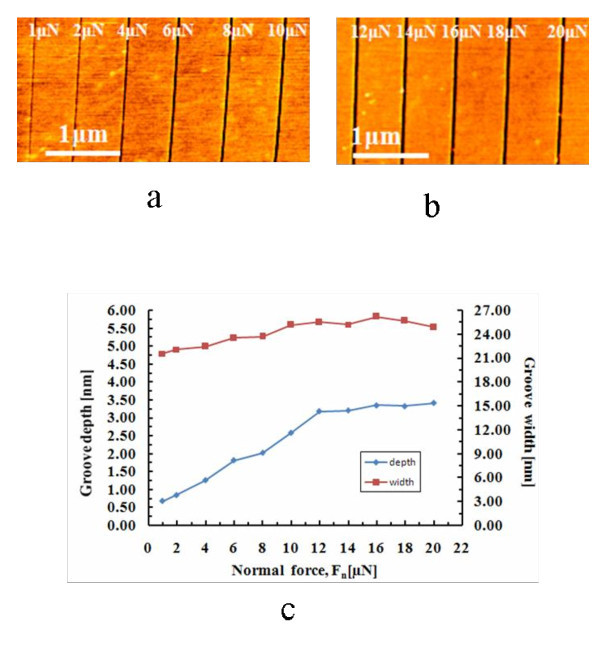
**AFM images of the grooves**. **(a-b) **The AFM images of the grooves scratched at 1 μm/s of scratch speed under different applied forces in the forward scratch. **(c) **The size of the scratched grooves as a function of the applied normal force.

The relationship between the groove size and the scratch parameters including applied normal force, number of scratch and scratch speed were further investigated in forward scratch. Ten cross-section profiles were randomly selected in different locations along the groove. The line width of the groove is defined by the full width at half maximum. The depth (*d*) and width (*W_f_*) of the groove were calculated from the measurement of the groove profiles at the ten points and were then averaged [28]. The AFM images of the scratched grooves generated under different normal forces are given in Figure 2a, b at both low-force regime (from approximately 0 to 10 μN) and high-force regime (from approximately 10 to 20 μN), respectively. The scratch was performed in the forward direction at one scratch cycle, and the scratch speed was fixed at 1 μm/s. The protuberances were observed along the banks near the groove mouth, which was caused mainly by plastic deformation during the scratch and was difficult to remove. The scratched groove size as a function of the applied normal force is shown in Figure 2c. With the increase of the applied normal force from 1 to 20 μN, the size of the grooves was increased from 0.68 to 3.35 nm in depth and from 21.59 to 26.19 nm in width. In the low-force regime, the groove depth and width increased linearly when the normal force ranging from 1 to 10 μN, while in the high-force regime from 12 to 20 μN, the groove depth and width increased slowly and the saturation characteristics occurred. Prioli *et. al. *reported similar phenomena on aluminum film by diamond tip [29]. This phenomenon indicating that the effective normal force markedly decreases when the contact area of the tip with the surface becomes larger at a force above 10 μN because of the nonlinear increments of the tip cross-section. Roughly speaking, the depth of the groove that can be scratched is proportional to the magnitude of the tip stresses (in which tip is acting as a cutting tool) that is the tip force divided by the lateral (horizontal) cross-section area of the tip. As shown in Figure 1a, the cross-section area increases much faster than the depth. Consequently, the nonlinear behavior of the relationship between groove dimension and tip force results from the nonlinear increments of the cross-section area of the tip.

**Figure 3:**
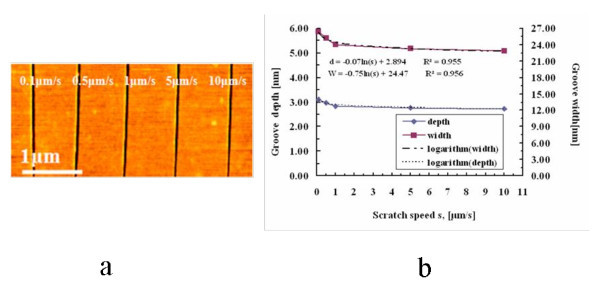
**AFM images of scratched grooves at different scratch speeds**. **(a) **The AFM images of the scratches at 10 μN of applied force in the forward scratch. **(b) **Correlation of the size of the scratched grooves with the scratch speed.

Experiments have been conducted to study the effect of changing the scratching speed on the shapes of scratched grooves. The AFM images of the scratched grooves at different scratch speeds are given in Figure 3a, and the corresponding depth and width of the grooves as a function of scratch speed is shown in Figure 3b. When the scratch speed increased from 0.1 to 10 μm/s, the depth decreased very slightly from 3.09 to 2.73 nm, indicating that the scratch speed did not have much influence on scratched depth. On the other hand, the width decreased sharply at low speed range and then reduced slowly at scratch speed higher than 1 μm/s, which fits a negative logarithm equation. However, the width changed from 26.36 to 22.9 nm and thus the total reduction was not significant, implying that AFM-based scratch nanolithography with a DLC-coated tip can be carried out at high scratch speed.

**Figure 4:**
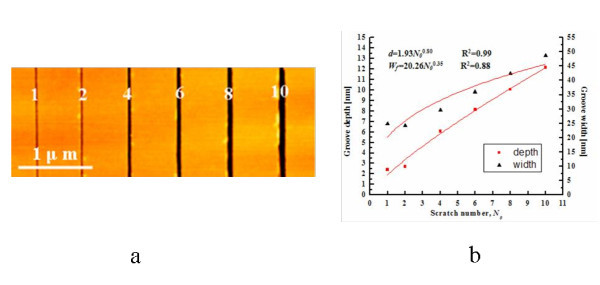
**Effects of the number of scratch or scan cycle**. **(a) **The AFM images of the scratches at 10 μN of applied force and 1 μm/s of scratch speed in the forward scratch. **(b) **Correlation of *d *and *W_f _*data with numbers of scratching cycle (*N*_o_) for Si(100).

Repeated scratches were also conducted in scratching experiments to study the effects of the number of the scratch or scan cycle on the size of the scratched geometry. It is expected that multiple or repeating scratch cycles along the same scratch path can enlarge the groove size. Figure 4a shows the AFM images of the grooves at different number of scratches. It was observed that small wear debris accumulating along the bank near the groove mouth during scratch. The corresponding depth and width of the grooves as a function of the number of scratches are given in Figure 4b. It was found that both depth and width increased linearly with the increase in the number of scratches. The size of the width increased faster than that of the depth. According to Ogino's report, the depth of the groove increased linearly, while the width of the groove was unchanged. The linear increase of the depth was attributed to the layer-by-layer removal mechanism during scratching.

Tseng *et al. *found that *d *and *W_f _*of the grooves increased with the number of scratch cycles (*N*_o_) following a power-law relationship [23]:

(1) $$d\left(N_{\text{o}}\right)=M_{1}{\left(N_{\text{o}}\right)}^{n_{1}}$$

and

(2) $$W_{f}\left(N_{\text{o}}\right)=M_{2}{\left(N_{\text{o}}\right)}^{n_{2}},$$

where *M_i _*and *n_i _*are the multiple scratch coefficient and multiple scratch exponent, respectively. As the *d *and *W_f _*data for multiple scratches on a Si(100) is illustrated in Figure 4b, the correlation values of *M*_1_, *M*_2_, *n*_1_, and *n*_2 _can be found to be 1.93, 20.26, 0.80, and 0.35 nm, respectively. The associated coefficient of determination (*R*^2^) is 0.99 for *d *and 0.88 for *W_f_*, which indicate that the power-law correlation fitting the depth data perfectly, and there is a 12% deviation for the width data.

**Figure 5:**
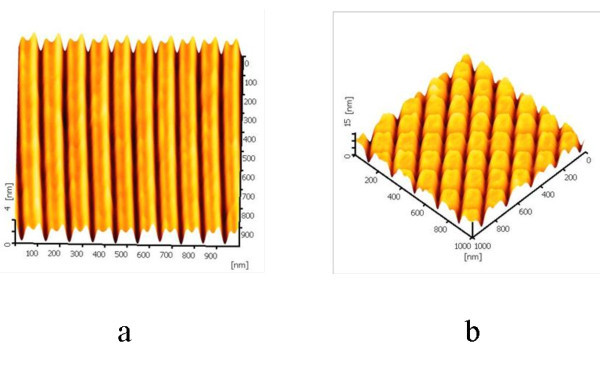
**AFM images of generated nanopatterns**. The AFM images of **(a) **the line arrays and **(b) **square arrays scratched at 10 μN of applied force, 1 μm/s of scratch speed, and four scratches.

Using a DLC-coated tip, more complex nanostructures including arrays of parallel lines and square arrays were fabricated by AFM scratch on Si substrate. Figure 5 shows the nanopatterns generated at 10 μN of the tip force, 1 μm/s of the scratch speed, and four scratches. For the arrays of parallel lines with an area of 1 × 1 μm^2^, the depth of the groove is about 2 nm with a pitch of 90 nm. As for the square arrays scratched on an area of 1 × 1 μm^2^, the depth of the groove is about 10 nm, and the dimension of a square area is 100 × 110 nm^2 ^with a pitch of 70 nm. The line arrays and square arrays in microscale were fabricated precisely and uniformly on a Si surface, indicating that AFM-based scratch lithography with a DLC-coated tip could be used to fabricate complex nanostructures on a hard silicon surface.

## Conclusion

In the present study, we explored the potential of the DLC-coated tip used as a cutting tool in AFM-based scratch nanolithography on a silicon surface. The scratched geometry was correlated to the scratch parameters, such as the scratch direction, applied tip force, scratch speed, and number of scratches. Uniform nanopatterns of line arrays and square arrays were further fabricated. This work provides an insight for fabricating nanopatterns on a hard material precisely and rapidly using an inexpensive AFM tip.

## Competing interests

The authors declare that they have no competing interests.

## Authors' contributions

XHJ and WGY are the primary authors and conceived of the study, carried out the experiments, characterization, acquisition of data, analysis and interpretation of data, drafting of the manuscript and revisions. And they contribute equally to this paper. JFZ and WSJ participated in language modification. AT participated in analysis and interpretation of data. ZLD is the principal investigator.
